# Diagnostic accuracy of glabellar tap sign for Parkinson’s disease

**DOI:** 10.1007/s00702-021-02391-3

**Published:** 2021-07-30

**Authors:** Simo Nuuttila, Mikael Eklund, Juho Joutsa, Elina Jaakkola, Elina Mäkinen, Emma A. Honkanen, Kari Lindholm, Tommi Noponen, Toni Ihalainen, Kirsi Murtomäki, Tanja Nojonen, Reeta Levo, Tuomas Mertsalmi, Filip Scheperjans, Valtteri Kaasinen

**Affiliations:** 1 Clinical Neurosciences, University of Turku, Turku, Finland; 2 Neurocenter, Turku University Hospital, Turku, Finland; 3 Turku PET Centre, Turku University Hospital, Turku, Finland; 4 Turku Brain and Mind Center, University of Turku, Turku, Finland; 5 Department of Psychiatry, Turku University Hospital, Turku, Finland; 6 Department of Clinical Physiology and Nuclear Medicine, Turku University Hospital, Turku, Finland; 7 Department of Medical Physics, Turku University Hospital, Turku, Finland; 8 HUS Medical Imaging Center, Clinical Physiology and Nuclear Medicine, University of Helsinki and Helsinki University Hospital, Helsinki, Finland; 9 Department of Neurology, Helsinki University Hospital, Helsinki, Finland; 10 Department of Clinical Neurosciences, University of Helsinki, Helsinki, Finland

**Keywords:** Parkinson’s disease, SPECT, Dopamine

## Abstract

Glabellar tap or reflex (GR) is an old bedside clinical test used in the diagnostics of Parkinson’s disease (PD), but its diagnostic value is unclear. This study examines the diagnostic validity and reliability of GR in PD in relation to brain dopaminergic activity. GR was performed on 161 patients with PD, 47 patients with essential tremor (ET) and 40 healthy controls immediately prior to dopamine transporter (DAT) [^123^I]FP-CIT SPECT scanning. The binding ratios were investigated with consideration of the GR result (normal/abnormal). In addition, the consistency of the GR was investigated with 89 patients after a mean follow-up of 2.2 years. PD and ET patients had higher GR scores than healthy controls (*p* < 0.001), but there was no difference in GR between PD and ET patients (*p* = 0.09). There were no differences in the ratio of abnormal to normal GRs between the PD and ET groups (73% vs. 64% abnormal, respectively, *p* = 0.13) or in DAT binding between PD patients with abnormal and normal GRs (*p* > 0.36). Over follow-up, the GR changed from abnormal to normal in 20% of PD patients despite the presence of clinically typical disease. The sensitivity and specificity of GR for differentiating PD from ET were 78.3% and 36.2%, respectively. Although GR has been used by clinicians in the diagnostics of PD, it does not separate PD from ET. It also shows considerable inconsistency over time, and abnormal GR has no relationship with dopamine loss. Its usefulness should be tested for other clinical diagnostic purposes.

## Introduction

The glabellar reflex (GR), also known as the glabellar tap sign, is an old clinical examination test first described by Dr. Walker Overend in 1896. No exact description of the maneuver has been established, but it is typically performed by the examiner gently tapping an index finger on the patient’s glabellar region, located between the eyebrows, after which a possible blink reflex is observed (Pearce et al. 1968). The traditional hypothesis states that healthy individuals quickly habituate to the stimulus, thus terminating the reflex after a few blinks or sometimes not blinking at all.

Overend did not describe in detail the mechanism underlying his hypothesis of the primitive reflex, but ever since it has been used by many neurologists as a simple diagnostic test for parkinsonian syndromes. Some neurologists have included the GR test in their routine neurological examination due to its potential value in supporting the diagnosis of Parkinson’s disease (PD). A number of studies have suggested that an abnormal test result may occur particularly in PD (Rushworth 1962; Jensen et al. 1983; Vreeling et al. 1993; Garland 1952), but an abnormal GR has also been described in numerous other conditions (Pearce et al. 1968; Jensen et al 1983; Thomas 1994). However, no research examining GR in relation to brain dopamine function—or any other biomarkers, for that matter—has been performed.

If an abnormal GR was associated with an abnormal PD-related biomarker, such as striatal dopamine transporter (DAT) binding, it would support its use in clinical practice. On the other hand, if the test had suboptimal specificity between degenerative and non-degenerative parkinsonisms, and no association with striatal DAT binding, it would question the rational use of GR as part of the clinical examination of a patient with suspected PD.

Previous research in this field has mainly been conducted with small samples, which could contribute to the inconsistency. A study with 100 participants indicated that further research with larger sample sizes was needed to examine the relationship between GR and parkinsonian disorders (Brodsky et al. 2004). Therefore, in this study, we investigated the relationship of GR and presynaptic striatal dopamine function and compared the results in a real-life sample of 248 patients with clinically unclear parkinsonian syndromes (CUPS), who were referred for diagnostic brain DAT imaging. In addition, a subsample of patients was re-examined after follow-up to investigate the reliability of GR over time.

## Methods

### Participants

**Fig. 1:**
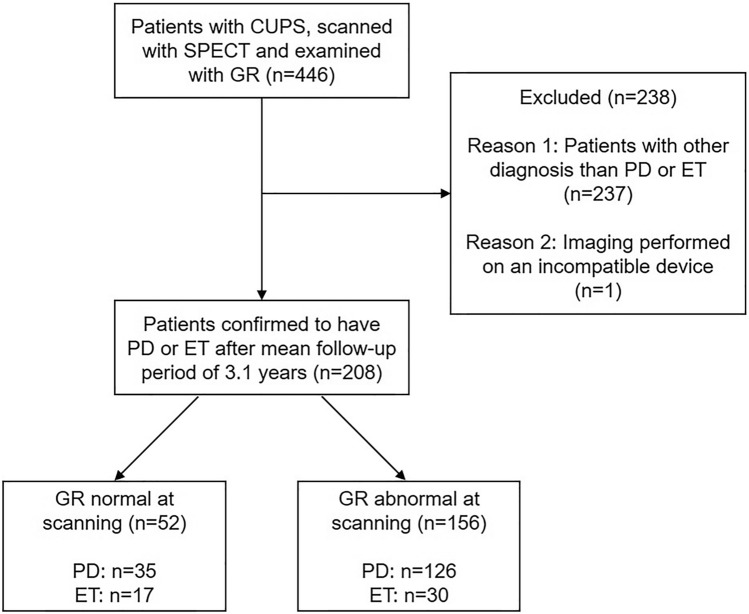
Flow of PD and ET patients. *CUPS* clinically unclear Parkinsonian syndrome

The flowchart of patients is presented in Fig. 1. The prospective study included 161 patients with PD, 47 patients with ET and 40 healthy controls. The GR test and dopamine transporter (DAT) [^123^I]FP-CIT SPECT were performed for each participant on the same day. The patients were clinically investigated 2–4 h before SPECT scanning (NMDAT study; ClinicalTrials.gov identifier: NCT02650843). In addition to GR, the examination included a clinical interview, the Unified Parkinson's Disease Rating Scale (MDS-UPDRS) part III, the Mini-Mental State Examination (MMSE) and the Beck Depression Inventory (BDI). All examiners successfully completed the MDS-UPDRS Training Program and Exercise. Patients with an MMSE score less than 18 were excluded from the study.

Patients with PD and ET were scanned with [^123^I]FP-CIT SPECT for clinical diagnostic purposes at Turku University Hospital or Helsinki University Hospital Medical Imaging Center, Finland, during the years 2014–2020. After a mean clinical follow-up period of 3.1 years (SD 1.5) after DAT imaging, the final clinical diagnoses of the patients (PD or ET) were established based on full patient histories, including medication response, symptoms, signs, laboratory results and imaging results.

Healthy controls were scanned using the same protocol, and each control participant underwent the same clinical examinations. To investigate the consistency of GR test results over time, 89 (PD *n* = 71, ET *n* = 18) patients were re-examined with GR and other clinical tests after a mean follow-up time of 2.2 years (SD 0.5) after scanning.

The study was approved by the Ethics Committee of the Turku Hospital district and was conducted according to the principles of the Declaration of Helsinki. Written informed consent was obtained from all participants included in the study.

### Glabellar reflex

Overend set the cutoff point for normality at three blinks, but varying cutoff points for a normal result have been suggested: the Simpson-Angus Scale drew the line at five blinks (Simpson and Angus 1970), whereas in another study, ten or fewer blinks was considered a normal result (Rao et al. 2003). We used a previously described protocol in which 21 consecutive, evenly paced taps were performed on the participant’s glabellar region with an approximate frequency of two taps per second (Simpson and Angus 1970). If no reflex was observed after three taps, the test was discontinued, and the result was considered normal. The examination was explained to the patient prior to its execution, and special attention was given to the positioning of the examiner and his or her index finger: the test was performed with the index finger pointing downwards and the examiner standing parallel to the patient to prevent the reflex from being evoked by a shadow or a visual threat. In accordance with the original description, twitching of the lower eyelids after the stimulus was registered, as was possible habituation to the stimulus (Overend 1896). Three twitches or fewer was considered a normal response and physiological habituation, whereas four twitches or more was considered an abnormal result.

### Imaging and image analysis

SPECT imaging started 3 h after the 185-MBq injection of [^123^I]FP-CIT. Potassium perchlorate (250–300 mg) or Jodix™ tablets (130 mg) were given 30–60 min before the injection to prevent exposure of the thyroid tissue to radiation. The SPECT data were acquired using one of our eight SPECT/CT devices. To allow data comparisons, all the SPECT/CT devices were calibrated using a striatal phantom (RSD, Radiology Support Devices, Inc., Long Beach, USA) before the study. The calibration procedure followed the guidance of Hermes Medical Solutions (Diemling 2012) and Tossici-Bolt et al. (2011). The SPECT data were reconstructed using the 3D OSEM algorithm with attenuation, collimator response and scatter corrections (HybridRecon Neurology, version 1.3, Hermes Medical Solutions AB, Stockholm, Sweden). The acquisition and reconstruction protocols were the same for all our devices and were based on EANM recommendations (Darcourt et al. 2010) Reconstructed images were analyzed using BRASS semiautomated analysis software (version 2.6, Hermes Medical Solutions, Stockholm, Sweden). Scanner-specific corrections based on our calibrations were used for the BRASS analyses (Diemling 2012; Tossici-Bolt et al. 2011; Varrone et al. 2013). Specific binding ratios (SBRs) for six regions (left and right caudate, anterior putamen and posterior putamen) were calculated using the occipital cortex as the reference region: SBR = (VOI _caudate or putamen_ − VOI _occipital_)/VOI _occipital_ (Varrone et al. 2013).

### Statistical analyses

For comparisons among three groups, ANOVA or Kruskal–Wallis tests were used, followed by pairwise testing with Tukey’s test or Bonferroni’s corrections. For comparisons between two groups, independent samples *t* tests, Mann–Whitney *U* tests or Chi-square tests were used. Analysis of covariance (ANCOVA), adjusted for motor symptom severity, age and scanning device, was used to compare groups of PD patients with normal and abnormal GR results. GR results and established clinical diagnosis were available for all patients. The level of statistical significance was set at *p* < 0.05. IBM SPSS Statistics (version 27, SPSS, Inc., Chicago, Illinois, USA) was used for all statistical analyses.

## Results

**Fig. 2:**
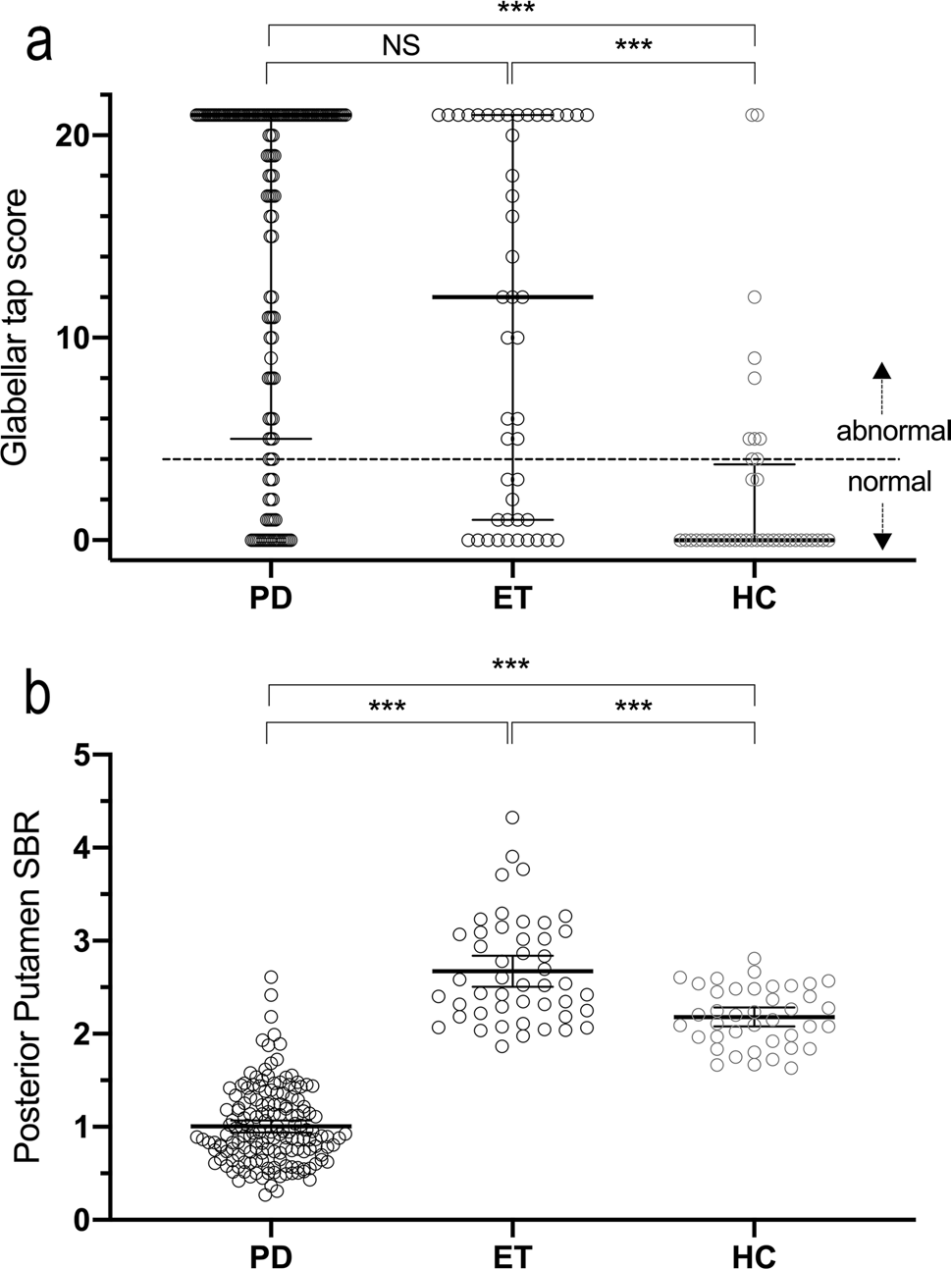
Glabellar tap score and specific binding ratio of the PD, ET and HC groups **a** Glabellar tap scores (range 0–21) of the PD, ET and HC groups, with the median and IQR marked with horizontal lines; note that in the PD patients, the median was 21 and thus is not visible **b** Mean posterior putamen specific binding ratio (SBR) of the PD, ET and HC groups, with the mean and 95% CI marked with horizontal lines ***p < 0.001

Demographic and clinical characteristics are presented in Table 1. Compared to the healthy individuals, both the PD and ET patients had higher (abnormal) GR scores (*p* < 0.001) (Fig. 2a). There were no differences between the PD and ET patients in GR scores (*p* = 0.09). There was no difference in the ratio of abnormal/normal GRs between the PD and ET patients (Table 1). The same was true whether the cutoff for abnormality was set at 5 (*p* = 0.14) or 10 (*p* = 0.09). Compared to the HC and ET patients, the PD patients had clearly lower striatal DAT binding values (62.4% lower mean posterior putamen SBR in PD vs. ET, *p* < 0.001) (Fig. 2b). A similar difference was observed for the caudate nucleus and anterior putamen (Table 1). The severity of motor symptoms in the PD patients and ET patients was comparable (*p* = 1.0), although the symptom duration in ET patients was longer (Table 1).

**Table 1:** Demographic and clinical characteristics of PD and ET patients compared to healthy individuals (HCs); values are means (SD), median [IQR], *n* or %

Variable group	Variable	PD	ET	HC	*p* value^1^ PD vs. ET vs. HC	*p* value^2^ PD vs. ET
Demographics	*n*	161	47	40	–	–
	Age (years)	64.5 (10.0)	65.4 (10.1)	66.8 (9.0)	0.41	1.0
	Sex (m/f)	81/80	25/22	19/21	0.87	1.0
Motor symptoms	MDS-UPDRS motor score	35.0 [22.0]	37.0 [59.0]	5.0 [21.0]	< 0.001	1.0
	Symptom duration (months)	27.6 [29.0]	73.4 [86.8]	NA	< 0.001	< 0.001
Cognition	MMSE	28.0 [3.0]	27.0 [3.0]	28.5 [3.0]	0.056	1.0
Depression	BDI	5.0 [9.0]	7.0 [9.0]	0.5 [5.0]	< 0.001	1.0
Glabellar reflex	Score	21.0 [16.0]	12.0 [20.0]	0.0 [4.0]	< 0.001	0.088
	Abnormal (*n*, %)	126, 78%	30, 64%	10, 25%	< 0.001	0.13
DAT binding	Caudate	2.14 (0.65)	3.11 (0.63)	2.58 (0.32)	< 0.001	< 0.001
	Anterior putamen	1.62 (0.57)	3.01 (0.65)	2.49 (0.33)	< 0.001	< 0.001
	Posterior putamen	1.00 (0.41)	2.67 (0.57)	2.18 (0.32)	< 0.001	< 0.001

^1^*p* values are from ANOVA, Chi-square test, Mann–Whitney *U* test or Kruskal–Wallis test

^2^*p* values are Bonferroni-corrected post hoc

The sensitivity and specificity of GR for differentiating between PD and ET were 78.3% and 36.2%, respectively. PD patients with an abnormal GR result had higher MDS-UPDRS motor scores and lower striatal DAT binding than PD patients with normal GR results (Table 2). When motor MDS-UPDRS, age and devices were used as covariates in the ANCOVA, there were no differences in DAT binding in any of the studied brain regions between patients with abnormal and normal GRs (*p* > 0.36). In both the ET and the HC group, there were no differences in DAT binding in any of the studied brain regions between those who had abnormal GR values and those with normal values (*p* > 0.21).

**Table 2:** Demographic and clinical characteristics of PD patients with a normal glabellar reflex (GR) result in relation to PD patients with an abnormal GR result; values are means (SD), medians [IQR] or *n*

Variable group	Variable	PD Normal GR	PD Abnormal GR	*p* value^1^
Demographics	*n*	35	126	–
	Age (years)	61.7 (10.7)	65.3 (9.8)	0.063
	Sex (m/f)	17/18	63/63	0.881
Motor symptoms	MDS-UPDRS motor score	29.0 [57.0]	38.5 [63.0]	0.004
	Symptom duration (months)	18.0 [24.0]	18.0 [26.0]	0.646
Cognition	MMSE	28.0 [3.0]	28.0 [4.0]	0.677
Depression	BDI	3.0 [7.0]	6.0 [8.0]	0.059
Glabellar reflex	Score	0.0 [1.0]	21.0 [3.0]	< 0.001
DAT binding	Caudate	2.36 (0.71)	2.08 (0.62)	0.021
	Anterior putamen	1.82 (0.63)	1.57 (0.55)	0.022
	Posterior putamen	1.15 (0.43)	0.96 (0.40)	0.014

^1^*p* values are from independent samples *t* test, Chi-square test or Mann–Whitney *U* test

In the follow-up cohort of 71 PD patients and 18 ET patients, the GR changed from abnormal to normal in 14 PD patients (20%) and in 7 ET patients (39%) over a mean period of 2.2 years. In 3 PD patients (4%), the GR changed from normal to abnormal over the same time period.

## Discussion

The present study shows that the GR test has suboptimal specificity for distinguishing PD from ET and no association with striatal DAT binding, questioning the rational use of GR as part of the clinical examination of a patient with suspected PD. Our study focused on PD and ET, two conditions that can be difficult to differentiate in early stages and whose differentiation is the official indication for clinical brain DAT imaging (FDA 2015; EMA 2017). The results show that (1) GR has suboptimal diagnostic value for PD with reference to ET and (2) when the effects of symptom severity and age are used as covariates, there is no relationship between GR and striatal DAT function in patients with PD, patients with ET or healthy controls.

The sensitivity and specificity of GR for differentiating between PD and ET were 78.3% and 36.2%, respectively, demonstrating that in clinical practice at bedside, GR produces a high number of both false positive and false negative findings. Previous studies have suggested that GR may be abnormal in individuals with other diseases and conditions, including Alzheimer’s disease, aging, hydrocephalus, HIV encephalopathy, schizophrenia, diffuse axonal injury, brain tumors, encephalitis, severe cerebral anoxia, presenile dementia and subarachnoid hemorrhagia (Pearce et al. 1968; Jensen et al. 1983; Thomas 1994; Formisano et al. 2009). Even in migraine, a marked proportion of patients may show no habituation in GR (Jensen et al. 1983). Our DAT findings further showed that there were no differences in presynaptic dopaminergic function between individuals with normal and abnormal GRs. This was also true for patients with PD when the effects of aging and motor symptom severity were controlled for. Therefore, abnormal GR appears to be associated with advanced disease stage and older age in PD, but it has no independent relationship with central dopamine loss. This is noteworthy, because DAT binding defect is a key pathophysiological event in PD, to the extent that a normal DAT scan is an exclusion criterion of PD (Postuma et al. 2015). It is important to note, however, that before correction for multiple comparisons, the difference in the abnormal/normal GT ratio between PD and ET was borderline significant, which suggests that there may be an underlying difference, albeit one that is insufficient for clinical diagnostic use. Indeed, in the follow-up segment of our study, we further observed considerable intraindividual variation in the GR, which underlines the ambiguity and lack of consistency of the GR as a diagnostic test.

If GR has no major clinical role in the differential diagnosis of degenerative parkinsonism, and if it is not linked to dopamine loss, the question remains regarding its mechanism and feasibility in clinical neurology. It is possible that blink reflex habituation and measurements have diagnostic value and clinical relevance for other conditions, such as primary dementing disorders (Mohammadian et al. 2015), headache syndromes (Avramidis et al. 2017), multiple sclerosis (Brooks et al. 2015), Bell’s palsy (Syed et al. 1999), or psychotic disorders (Taiminen et al. 2000). However, further studies with large samples in these diagnostic groups are needed.

One should note that MDS-UPDRS motor scores were high also in our ET patients suggesting that ET phenotypes were in many cases ET plus (Bhatia et al. 2018) which could be pathophysiologically different as compared to typical cases of purely tremolous ET. In addition, the high scores of ET patients underline the potential phenotypic overlap of ET and PD. In our study the concept of overlap is also supported by equal GR scores in both groups (*p* = 0.09) and high BDI scores of both PD and ET patients compared to healthy controls (*p* < 0.001). The differential diagnostics of the two conditions on a clinical basis can be very challenging, and the clinical picture of both conditions can resemble that of the other at different stages of the illness (Algarni and Fasano 2017).

It is also of relevance to note that, in PD, previous kinematic studies have documented normal velocity and amplitude of the closing and opening phases during voluntary blinking, but increased duration of the pause between the opening and closing phases (inter-phase pause) (Agostino et al. 2008). On the other hand, during reflex blinking, the kinematic parameters or the length of the inter-phase pause appear to be similar in PD patients and healthy controls (Agostino et al. 2008). These and other findings indicate that the brainstem circuits of the blink reflex are generally preserved in PD (Bologna et al. 2013), and the miscoordination of the timing and reciprocity of the muscles required for blinking are primarily caused by the impaired basal ganglia and interconnected cortical structures in PD (Agostino et al. 2008). The role of the basal ganglia is supported by evidence suggesting that spontaneous blinking can be increased with subthalamic nucleus deep brain stimulation (Bologna et al. 2012) and dopaminergic replacement therapy (Karson 1983).

The reliability of our results is supported by the large sample size, the use of DAT imaging and the clinical follow-up. The study is limited by the lack of neuropathological diagnostic verification, a common problem in most PD neuroimaging studies, and the use of several scanning sites with different SPECT/CT devices. However, the devices were intercalibrated, and the results were similarly nonsignificant whether device was or was not included as a covariate in the analysis. The somewhat higher DAT binding in ET patients compared to healthy controls could be associated with a self-selection bias in the control group (patients with mild subclinical parkinsonian symptoms might more likely volunteer to participate) and/or a selection bias in the ET group (patients with mild DAT defects were excluded). The difference, however, had no effect on the primary results.

In summary, the present study shows that the accuracy of the glabellar tap test in the diagnosis of PD is suboptimal, and the test results are inconsistent over time. The usefulness of the GR should be tested for other clinical diagnostic purposes, but it does not seem to serve purpose in the clinical diagnosis of PD.

## Data Availability

Data not provided in the article will be shared at the request of other investigators for purposes of replicating procedures and results.

## References

[CR1] Agostino R, Bologna M, Dinapoli L, Greogri B, Fabbrini G, Accornero N, Berardelli A (2008). Voluntary, spontaneous, and reflex blinking in Parkinson’s disease. Mov Disord.

[CR2] Algarni M, Fasano A (2017). The overlap between Essential tremor and Parkinson disease. Parkinsonism Relat Disord.

[CR3] Avramidis T, Bougea A, Hadjigeorgiou G, Thomaides T, Papadimitriou A (2017). Blink reflex habituation in migraine and chronic tension-type headache. Neurol Sci.

[CR4] Bhatia KP, Bain P, Bajaj N, Elble RJ, Hallett M, Louis ED, Raethjen J, Stamelou M, Testa CM, Deuschl G (2018). Tremor task force of the international Parkinson and movement disorder society. Consensus statement of the classification of tremors. Mov Disord.

[CR5] Bologna M, Fasano A, Modugno N, Fabbrini G, Berardelli A (2012). Effects of subthalamic nucleus deep brain stimulation and l-dopa on blinking in Parkinson’s disease. Exp Neurol.

[CR6] Bologna M, Fabbrini G, Marsili L, Defazio G, Thompson PD, Berardelli A (2013). Facial bradykinesia. J Neurol Neurosurg Psychiatry.

[CR7] Brodsky H, Dat Vuong K, Thomas M, Jankovic J (2004). Glabellar and palmomental reflexes in Parkinsonian disorders. Neurology.

[CR8] Brooks JB, Jardim MR, Papais-Alvarenga RM, Fragoso YD (2015). There is still a role for the blink reflex in the diagnosis and follow-up of multiple sclerosis. Clin Neurophysiol.

[CR9] Darcourt J, Booij J, Tatsch K, Varrone A, Vander Borght T, Kapucu OL, Någren K, Nobili F, Walker Z, Van Laere K (2010). EANM procedure guidelines for brain neurotransmission SPECT using (123)I-labelled dopamine transporter ligands, version 2. Eur J Nucl Med Mol Imaging.

[CR10] Diemling M (2012) Instructions on implementation of phantom based camera corrections for Enc-Dat BRASS. 14.1.2012: Hermes Medical Solutions; 2012

[CR11] European Medicines Agency (EMA) (2017) Product information for DatScan Ioflupane I 123 Injection. https://www.ema.europa.eu/en/documents/product-information/datscan-epar-product-information_en.pdf2017. Accessed 14 Feb 2021

[CR12] Food and Drug Administration (FDA) (2015) Highlights of prescribing information for DatScan Ioflupane I 123 injection. https://www.accessdata.fda.gov/drugsatfda_docs/label/2015/022454Orig1s004lbl.pdf2015. Accessed 14 Feb 2021

[CR13] Formisano R, Cicinelli P, Buzzi MG, Brunelli S, Zafonte R, Vinicola C, Gabrielli A, Sabatini U (2009). Blink reflex changes in parkinsonism following severe traumatic brain injury correlates with diffuse axonal injury. Med Sci Monit.

[CR14] Garland HG (1952). Parkinsonism. Br Med J.

[CR15] Jensen JP, Grøn U, Pakkenberg H (1983). Comparison of three primitive reflexes in neurological patients and in normal individuals. J Neurol Neurosurg Psychiatry.

[CR16] Karson CN (1983). Spontaneous eye-blink rates and dopaminergic systems. Brain.

[CR17] Mohammadian F, Noroozian M, Nafissi S, Fatehi F (2015). Blink reflex may help discriminate Alzheimer disease from vascular dementia. J Clin Neurophysiol.

[CR18] Overend W (1896). Preliminary note on a new cranial reflex. The Lancet.

[CR19] Pearce J, Aziz H, Gallagher JC (1968). Primitive reflex activity in primary and symptomatic Parkinsonism. J Neurol Neurosurg Psychiatry.

[CR20] Postuma RB, Berg D, Stern M, Poewe W, Olanow CW, Oertel W, Obeso J, Marek K, Litvan I, Lang AE, Halliday G, Goetz CG, Gasser T, Dubois B, Chan P, Bloem BR, Adler CH, Deuschl G (2015). MDS clinical diagnostic criteria for Parkinson's disease. Mov Disord.

[CR21] Rao G, Fisch L, Srinivasan S, D'Amico F, Okada T, Eaton C, Robbins C (2003). Does this patient have Parkinson disease?. JAMA.

[CR22] Rushworth G (1962). Observations on blink reflexes. J Neurol Neurosurg Psychiatry.

[CR23] Simpson GM, Angus JW (1970). A rating scale for extrapyramidal side effects. Acta Psychiatr Scand Suppl.

[CR24] Syed NA, Delgado A, Sandbrink F, Schulman AE, Hallett M, Floeter MK (1999). Blink reflex recovery in facial weakness: an electrophysiologic study of adaptive changes. Neurology.

[CR25] Taiminen T, Jääskeläinen S, Ilonen T, Meyer H, Karlsson H, Lauerma H, Leinonen KM, Wallenius E, Kaljonen A, Salokangas RK (2000). Habituation of the blink reflex in first-episode schizophrenia, psychotic depression and non-psychotic depression. Schizophr Res.

[CR26] Thomas RJ (1994). Blinking and the release reflexes: are they clinically useful?. J Am Geriatr Soc.

[CR27] Tossici-Bolt L, Dickson JC, Sera T, de Nijs R, Bagnara MC, Jonsson C, Scheepers E, Zito F, Seese A, Koulibaly PM, Kapucu OL, Koole M, Raith M, George J, Lonsdale MN, Münzing W, Tatsch K, Varrone A (2011). Calibration of gamma camera systems for a multicentre European ^123^I-FP-CIT SPECT normal database. Eur J Nucl Med Mol Imaging.

[CR28] Varrone A, Dickson JC, Tossici-Bolt L, Sera T, Asenbaum S, Booij J, Kapucu OL, Kluge A, Knudsen GM, Koulibaly PM, Nobili F, Pagani M, Sabri O, Vander Borght T, Van Laere K, Tatsch K (2013). European multicentre database of healthy controls for [123I]FP-CIT SPECT (ENC-DAT): age-related effects, gender differences and evaluation of different methods of analysis. Eur J Nucl Med Mol Imaging.

[CR29] Vreeling FW, Verhey FR, Houx PJ, Jolles J (1993). Primitive reflexes in Parkinson's disease. J Neurol Neurosurg Psychiatry.

